# Is cytochrome modulation the new frontier for decreasing the risk of cataract?

**DOI:** 10.4103/0253-7613.51344

**Published:** 2009-04

**Authors:** Kavitha S. Nair, Kirti V. Patel, Tejal R. Gandhi

**Affiliations:** Department of Pharmacology, Anand Pharmacy College, Opp Town Hall, Anand - 388 001, Gujarat, India

**Keywords:** Cataract, cytochrome P450, diltiazem, pioglitazone

## Abstract

**Aim:**

The present study was designed to study the effect of cytochrome P450 (CYP) modulators on the occurrence of cataract using male Sprague-Dawley rats weighing 40:50 gm.

**Materials and Methods:**

Macroscopical examination of the lens isolated from rats pretreated with diltiazem (30 mg/kg; once daily; PO) showed delayed occurrence of cataract while pioglitazone (3.8 mg/kg; once daily; PO) pretreatment demonstrated an early cataract.

**Results and Conclusion:**

A delayed occurrence of cataract with diltiazem (CYP inhibitor) and an early onset of cataract with pioglitazone (CYP inducer) indicate that a cytochrome P450 mediated pathway may affect the initiation of cataract but not the maturation pattern.

## Introduction

Cataract is clouding of the eye lens that reduces the amount of incoming light and results in deteriorating vision. Cataract remains the leading cause of visual disability and blindness all over the globe making up at least 50% of blindness in most developing countries.[1] Blindness is thought to reach 75 million by 2020. Of these, unoperated cataract may be expected to account for at least 35 million. This figure is equivalent to the combined present populations of Australia, New Zealand, Sweden and Denmark. Thus, the burden of cataract is increasing remorselessly.[1]

Increased incidence of cataracts in diabetic patients is also well known. Excess glucose is converted to sorbitol by the enzyme aldose reductase using NADPH as a cofactor. Electron transfer from NADPH further depends on cytochrome P450 system. A typical cytochrome P450 catalyzed reaction is:

NADPH + H^+^ + O_2_ + RH == > NADP^+^ + H_2_O + R-OH

Thus, we hypothesized that by inducing or inhibiting CYP one can alter the activity of aldose reductase and thus the formation of sorbitol and therefore the occurrence of cataract.

In addition to diabetes, ageing is also a major risk factor for cataract. Moreover, a geriatric population suffers from a variety of diseases such as hypertension, diabetes etc. Such patients are often treated with multiple drugs, some of which are cytochrome P450 (CYP) modulators. Thus by simply altering the medicines (i.e., preferring CYP inhibitors instead of CYP inducers) one can reduce the risk of occurrence of cataract. Therefore the present study was undertaken to evaluate the effect of a CYP inducer (pioglitazone)[2] and a CYP inhibitor (diltiazem)[3] on the occurrence of cataract using the galactose induced cataract model.

## Materials and Methods

Male Sprague-Dawley rats weighing 40-50 gm were randomly divided into four groups, with six animals in each group. The normal control group (group I) was fed with laboratory chow. Cataract was induced in groups II, III and IV by feeding a galactose rich diet[4] starting from day 23 after parturition. Additionally group III was pretreated with diltiazem (30 mg/kg; once daily; PO) and group IV with pioglitazone (3.8 mg/kg; once daily; PO) starting on day 18 after parturition.

All animals were checked daily for the appearance of cataract with an ophthalmoscope (OM -18, Takagi Resolution 1.6). The experiment was continued until all the lenses were affected with cataract.

### Statistical analysis

Statistical significance was determined by ANOVA followed by Tukey's test.

## Results

**Figure 1:**
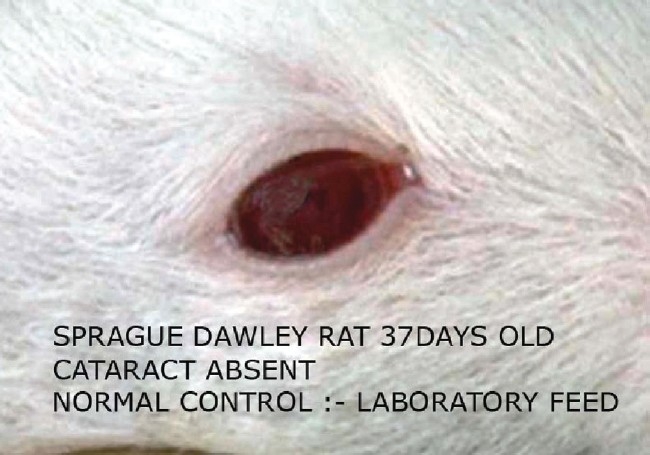
Normal control cataract absent

**Figure 2:**
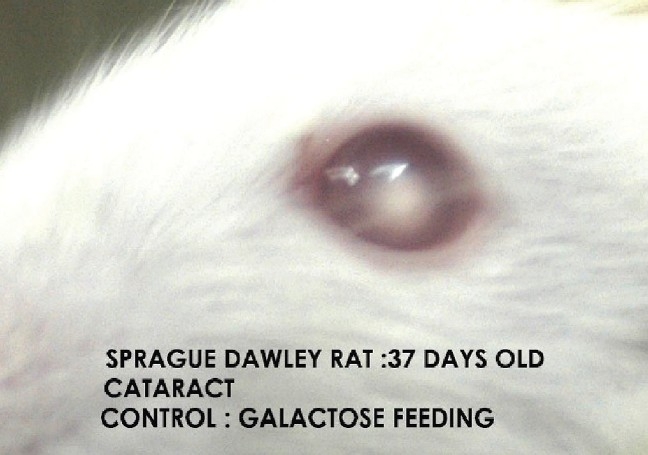
Cataract model control/ group II galactose feeding

**Figure 3:**
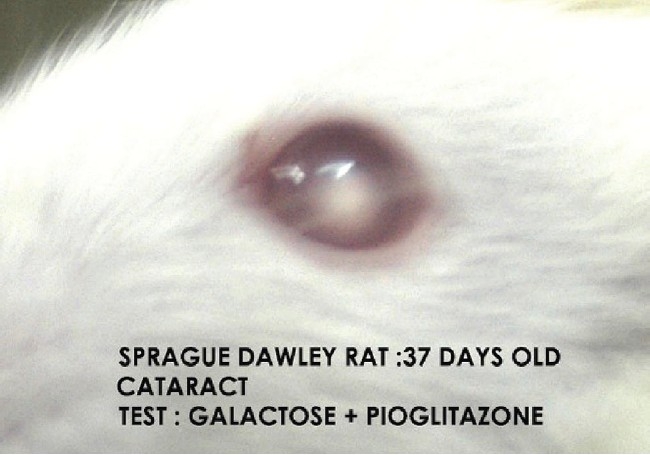
Cataract group IV/test group galactose+pioglitazone pretreated

**Figure 4:**
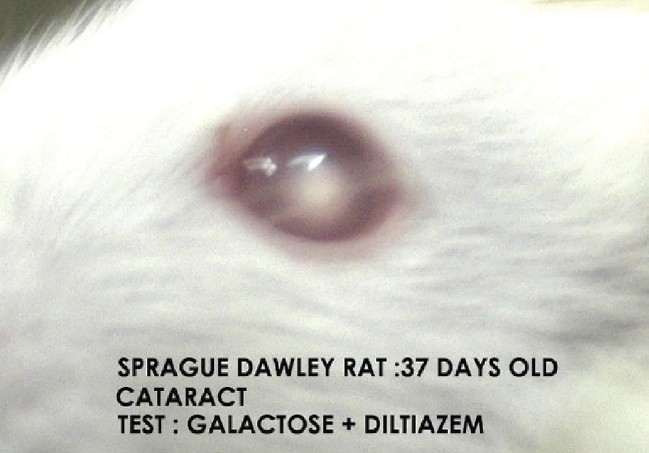
Cataract group III/test group galactose+diltiazem pretreated

In the present study cataract was absent in the normal control group [Figure 1]. Macroscopical examination of the lenses of the animals fed on the galactose diet showed the development of cataract (100% of lens) after day 14 of galactose feeding [Table 1, Figure 2]. In the diltiazem pretreated group, cataract formation was seen only in 8.3% of lenses on day 12 against 16.6% of the lenses in galactose control group demonstrating a significant (*P* ≤ 0.05) delay in cataract [Table 1]. On the other hand pioglitazone pretreatment demonstrated a significant (*P* ≤ 0.05) early cataract as evidenced by 8.3% of cataractous lenses on day 10 of galactose feeding when compared with 0% of cataractous lenses in the galactose control animals [Table 1]. Furthermore, the maturation pattern was comparable in both test groups viz., pioglitazone [Figure 3] pretreated and diltiazem [Figure 4] pretreated, reflected as 100% of the lens being affected on day 18 (i.e. 37th day of life) in both the groups.

**Table 1:** Effect of diltiazem and pioglitazone on the progression of cataract

*nth day of galactose administration*	*Galactose control (% lens affected)#*	*Pioglitazone pretreated (% lens affected)#*		*Diltiazem pretreated (% lens affected)#*
1	0	0		0
2	0	0		0
3	0	0		0
4	0	0		0
5	0	0		0
6	0	0		0
7	0	0		0
8	0	0		0
9	0	0		0
10	0	8.3*		0*
11	0	8.3*	\	0*
12	16.6	33.3*		8.3*
13	41.6	33.3*		16.6*
14	100	33.3*		25*
15	100	41.6*		50*
16	100	66.6*		58.3*
17	100	75*		58.3*
18	100	100		100

* - statistically significant from galactose control and *P* ≤ 0.05; Statistical significance was determined by ANOVA followed by Tukey;

# - % lens affected is in terms of total number of lenses affected

## Discussion

Increased incidence of cataracts in diabetic patients is well known. Evidence has accumulated for the involvement of polyol metabolism and the enzyme aldose reductase in diabetic cataractogenesis.[5,6] Sugar (galactose)-induced cataractogenesis in rats has been shown to parallel lenticular polyol accumulation.[7] The enzyme aldose reductase catalyzes the reduction of galactose to the corresponding polyols, i.e., dulcitol. The formation of polyols (in sugar cataract) by aldose reductase requires NADPH as a cofactor which is dependent on cytochromes for electron transfer. Since polyols do not readily diffuse through intact cellular membranes, they create a severe osmotic stress within the lenticular cells which leads to cellular swelling and loss of integrity of the cellular membrane.[8]

This implies that, by inhibiting or inducing cytochromes one can regulate the activity of aldose reductase via inhibition or induction of NADPH electron transfer and hence the occurrence of cataract. Similarly in the present study macroscopical examination of the lenses of the animals fed on the galactose diet showed the development of cataract (100% of lens) after day 14 of galactose feeding. In the diltiazem pretreated group, cataract formation was seen in only 8.3% of lenses on day 12 against 16.6% of the lenses in galactose control group demonstrating a significant (*P* ≤ 0.05) delay in cataract. On the other hand pioglitazone pretreatment demonstrated a significant (*P* ≤ 0.05) early cataract as evidenced by 8.3% of cataractous lenses on day 10 of galactose feeding when compared with 0% of cataractous lenses in the galactose control animals. Furthermore, the maturation pattern was comparable in both test groups viz., pioglitazone pretreated and diltiazem pretreated reflected as 100% of the lens being affected on day 18 in both the groups. Thus, it can be concluded that the drugs viz., diltiazem (30 mg/kg; once daily; PO) and pioglitazone (3.8 mg/kg; once daily; PO) affected the initiation of cataract via a cytochrome P450 mediated pathway but not the maturation pattern. This unleashes a novel insight into the role played by cytochrome P450 for decreasing the risk of cataract. Therefore, it can be concluded that by inducing or inhibiting CYP one can alter the activity of aldose reductase and thus the formation of sorbitol and therefore the occurrence of cataract. However, it has been reported that the extent of CYP isozyme induction or inhibition increases significantly as the dose of the drug increases and/or duration of treatment increases.[9] Thus, further studies need to be done to evaluate the efficacy in varied doses, as this is only a single dose study.
